# Linear association between high-sensitivity C-reactive protein and postoperative delirium after general anesthesia: a cross-sectional study

**DOI:** 10.3389/fneur.2025.1516800

**Published:** 2025-01-31

**Authors:** Xiao Qin, Junming Ren, Chunping Xing, Lijiao Chen, Renjie Wang, Shouyuan Tian

**Affiliations:** ^1^Department of Anesthesiology, Sixth Hospital of Shanxi Medical University, General Hospital of Tisco, Taiyuan, China; ^2^Department of Anesthesiology, Shanxi Provincial People's Hospital, Taiyuan, China; ^3^Department of Anesthesiology, Ruijin Hospital, Shanghai Jiao Tong University School of Medicine, Shanghai, China; ^4^Department of Urology, General Hospital of Tisco, Sixth Hospital of Shanxi Medical University, Taiyuan, China; ^5^Shanxi Province Cancer Hospital, Shanxi Hospital Affiliated to Cancer Hospital, Chinese Academy of Medical Sciences, Cancer Hospital Affiliated to Shanxi Medical University, Taiyuan, China

**Keywords:** inflammation, high-sensitivity C-reactive protein, postoperative delirium, general anesthesia, restricted cubic spline

## Abstract

**Objective:**

To investigate the association between high-sensitivity C-reactive protein (Hs-CRP) levels and the risk of postoperative delirium (POD) following general anesthesia.

**Methods:**

This retrospective cross-sectional study included 644 patients who underwent general anesthesia. Univariate and multivariate logistic regression analyses were performed to evaluate the relationship between Hs-CRP and POD, with subgroup analyses used to assess stratified associations. Receiver operator characteristic (ROC) curve analysis was employed to assess the predictive efficacy of Hs-CRP for POD. Restricted cubic spline (RCS) analysis was conducted to explore the linear relationship between the log-transformed Hs-CRP (Log_10_Hs-CRP) and POD risk.

**Results:**

The total population consisted of 644 individuals with a mean age of 64.02 ± 13.20 years, 506 (78.60%) of whom were male, and 114 patients (17.7%) had POD. Compared to the lower Hs-CRP group, patients in the higher Hs-CRP group exhibited higher age, heart rate, white blood cell count, blood urea nitrogen, creatinine, uric acid, fasting glucose, hemoglobin A1c, fibrinogen, D-dimer, and a higher prevalence of CKD, but lower hemoglobin, high-density lipoprotein cholesterol, albumin and estimated glomerular filtration rate. Additionally, the prevalence of POD was higher in the higher Hs-CRP group (24.7% vs. 9.5%, *p* < 0.001). Multivariate logistic regression confirmed that elevated Hs-CRP and its forms (Log_10_Hs-CRP, standardized Hs-CRP, and higher Hs-CRP group) consistently increased the risk of POD across all adjusted models (*p* < 0.05). Stratified analyses further highlighted significant associations between Hs-CRP and POD in specific subgroups, notably in patients aged ≥65 years, female patients, and those with or without hypertension, diabetes, or stroke history, and without chronic kidney disease (*p* < 0.05). ROC curve analysis demonstrated that Hs-CRP had a significant predictive ability for POD in the overall population (AUC = 0.646), as well as in male (AUC = 0.644) and female patients (AUC = 0.654). Additionally, RCS analysis indicated a linear positive association between Log_10_Hs-CRP and POD risk (*p* = 0.003, nonlinear *p* = 0.896).

**Conclusion:**

Elevated Hs-CRP levels are significantly associated with an increased risk of POD following general anesthesia.

## 1 Introduction

Postoperative delirium (POD) is a common postoperative complication, particularly prevalent among elderly patients and those undergoing surgery with general anesthesia (1). POD typically manifests as confusion, inattention, and cognitive impairment, which not only hinders patient recovery but may also extend hospital stays, increase healthcare costs, and even elevate mortality rates (2–4). Therefore, identifying risk factors for POD and implementing early interventions is crucial for improving patient outcomes.

In recent years, the inflammatory response has been recognized as a key factor in the development of POD (5). Studies have shown that surgery and anesthesia can activate the inflammatory response, leading to the release of various inflammatory mediators, which may, in turn, affect the central nervous system (6, 7). High-sensitivity C-reactive protein (Hs-CRP), a sensitive marker of inflammation, is elevated in numerous acute and chronic inflammatory states (8). Hs-CRP is not only used to assess postoperative infection risk but is also considered to be associated with neuroinflammatory processes in certain neurological disorders (9, 10). Therefore, some researchers have begun to focus on the association between inflammation and POD. For example, several studies have indicated a close relationship between CRP and POD following surgery with general anesthesia in various populations (11–13). However, the correlation between Hs-CRP and POD in the context of surgery with general anesthesia has not yet been fully explored.

Therefore, to further clarify the relationship between Hs-CRP and POD risk, this study adopts a cross-sectional design to evaluate the association between Hs-CRP levels and POD occurrence in patients undergoing surgery with general anesthesia, aiming to provide new insights for the early prediction and intervention of POD.

## 2 Methods

### 2.1 Study population

This study received approval from the Ethics Committee of the Sixth Hospital of Shanxi Medical University, and all procedures strictly adhered to the ethical principles outlined in the Declaration of Helsinki to ensure the safety and rights of the study subjects. As this study was retrospective, utilizing data from previous medical records that have been de-identified, informed consent from patients was waived. This study was a cross-sectional analysis involving patients who underwent general anesthesia at the Sixth Hospital of Shanxi Medical University from January 2019 to January 2024. A total of 644 patients who met the inclusion criteria were enrolled. The inclusion criteria were as follows: patients aged ≥18 years who underwent general anesthesia for elective non-cardiac surgeries, such as general surgery, orthopedics, burns, and urology, and received a POD assessment. The exclusion criteria were: (1) patients with a history of severe mental illness or cognitive impairment; (2) patients who died due to intraoperative or postoperative complications; (3) patients who used medications prior to surgery that could affect the assessment of delirium (such as antipsychotics or sedatives); (4) patients with acute or chronic infections, malignant tumors, or other conditions that could significantly influence Hs-CRP levels; (5) patients who underwent surgery with cardiopulmonary bypass or experienced severe hypoxic events; (6) patients who were unable to complete the POD assessment during the study period; (7) patients with missing baseline Hs-CRP data; and (8) patients with other major missing baseline data.

### 2.2 Diagnosis of postoperative delirium

In this study, POD was diagnosed using the Confusion Assessment Method (CAM), a widely used clinical tool for rapid screening. CAM diagnoses delirium by assessing patients based on four criteria: acute onset and fluctuating course, inattention, disorganized thinking, and altered level of consciousness. Specifically, if a patient meets the first two criteria and at least one of the latter two (disorganized thinking or altered level of consciousness), they are diagnosed with delirium (14). Due to its high sensitivity and specificity, CAM is recommended for the rapid assessment of postoperative patients and is simple to use, making it suitable for administration by trained healthcare professionals (15).

### 2.3 Covariate collection and assessment

This study collected and assessed various covariates, including demographic information (age and gender), lifestyle factors (smoking and drinking), comorbidities, biomarkers, and vital signs. Smoking status was defined as smoking at least one cigarette daily within the past year. Alcohol consumption was defined as consuming alcohol at least once per week within the past year. Comorbidity information included hypertension, diabetes, stroke history, and chronic kidney disease (CKD). Hypertension was defined based on medical history, with systolic blood pressure (SBP) ≥ 140 mmHg and/or diastolic blood pressure (DBP) ≥ 90 mmHg or current antihypertensive treatment (16). Diabetes was defined by medical history, glycated hemoglobin (HbA1c) level ≥ 6.5%, fasting glucose ≥7.0 mmol/L, or prior diagnosis and treatment (17). Stroke history included any type of stroke (such as ischemic or hemorrhagic) documented in the medical records. CKD was defined by an estimated glomerular filtration rate (eGFR) < 60 mL/min/1.73 m^2^ or a prior diagnosis of CKD (18). The eGFR was calculated using the CKD-EPI equation, which includes serum creatinine levels, age, and gender, to provide an accurate assessment of kidney function (19).

Body mass index (BMI) was calculated based on height and weight measurements, expressed in kg/m^2^. Vital signs, including SBP, DBP, and heart rate, were measured preoperatively with an automated blood pressure monitor following standardized protocols. Blood samples were also collected preoperatively to measure biomarkers, including hemoglobin, white blood cell count, platelet count, uric acid, creatinine, blood urea nitrogen (BUN), total cholesterol, low-density lipoprotein cholesterol (LDL-C), high-density lipoprotein cholesterol (HDL-C), triglycerides, alanine aminotransferase (ALT), aspartate aminotransferase (AST), total bilirubin, albumin, fibrinogen, D-dimer, fasting glucose, HbA1c and Hs-CRP. Hs-CRP levels were categorized into two groups based on the optimal cutoff value of 4.78 mg/L determined through receiver operating characteristic (ROC) curve analysis: lower Hs-CRP (Hs-CRP ≤ 4.78 mg/L) and higher Hs-CRP (Hs-CRP > 4.78 mg/L). All biomarkers, including Hs-CRP, were collected during routine preoperative examinations before surgery and sent to a clinical laboratory for measurement using standardized operating procedures and certified equipment to ensure accuracy and reproducibility.

### 2.4 Statistical methods

All statistical analyses were performed using SPSS 26.0, MedCalc 19.6.1, and R 4.1.3 software. Continuous variables were expressed as mean ± standard deviation or median (interquartile range), depending on the data distribution, and were compared between groups using the independent sample t-test or the Mann–Whitney U test. Categorical variables were presented as frequencies and percentages, and comparisons were made using the chi-square test or Fisher’s exact test. To assess the association between Hs-CRP and the risk of POD, univariate logistic regression analysis was initially conducted to calculate the odds ratios (OR) and their 95% confidence intervals (CI) for each variable. Subsequently, multivariable logistic regression analysis was performed with three different adjustment models: Model 1 adjusted for age and gender; Model 2 further adjusted for smoking, drinking, hypertension, diabetes, stroke, and CKD; and Model 3 included additional adjustments for SBP, DBP, hemoglobin, AST, total bilirubin, albumin, BUN, creatinine, eGFR, uric acid, HbA1c, and fibrinogen. To further explore the relationship between Hs-CRP and the risk of POD, we conducted stratified analysis and restricted cubic spline (RCS) analysis. Stratified analysis was performed based on age, gender, hypertension, diabetes, stroke history, and CKD, with adjustments for other variables to evaluate the association between Hs-CRP and POD within different subgroups. RCS analysis was used to evaluate the linear relationship between Log_10_Hs-CRP and the risk of POD and to determine the nonlinear *p* value to assess the significance of any nonlinear associations. Finally, receiver operating characteristic (ROC) curve analysis was conducted to assess the accuracy of Hs-CRP in predicting POD in different gender groups and the overall population, with the area under the curve (AUC) and its 95% CI calculated. All statistical analyses were considered significant at a two-sided *p* < 0.05 level.

## 3 Results

### 3.1 Clinical data grouped by high-sensitivity C-reactive protein

As shown in Table 1, the total population consisted of 644 individuals with a mean age of 64.02 ± 13.20 years, 506 (78.60%) of whom were male, and 114 patients (17.7%) had POD. patients in the higher Hs-CRP group exhibited significant differences across several clinical indicators. Firstly, age was notably higher in this group (65.25 vs. 62.56 years, *p* = 0.010). Compared to the lower Hs-CRP group, the higher Hs-CRP group had a higher prevalence of CKD (26.4 vs. 10.1%, *p* < 0.001), elevated heart rate (85.11 vs. 79.26 bpm, *p* < 0.001), increased white blood cell count (10.91 vs. 9.08 ×10^9^/L, *p* < 0.001), BUN (6.15 vs. 5.30 mmol/L, *p* < 0.001), creatinine (84.00 vs. 73.50 μmol/L, *p* < 0.001), uric acid (374.57 vs. 340.60 μmol/L, *p* < 0.001), fasting glucose (6.56 vs. 6.13 mmol/L, *p* = 0.012), HbA1c (6.85 vs. 6.46%, *p* = 0.012), fibrinogen (4.27 vs. 3.37 g/L, *p* < 0.001), and D-dimer (217.50 vs. 145.00 μg/L, *p* < 0.001). However, levels of hemoglobin (132.53 vs. 142.18 g/L, *p* < 0.001), HDL-C (1.10 vs. 1.18 mmol/L, *p* < 0.001), albumin (36.88 vs. 38.93 g/L, *p* < 0.001), and eGFR (83.86 vs. 103.67 mL/min/1.73 m^2^, *p* < 0.001) were significantly lower. Additionally, the prevalence of POD was higher in the higher Hs-CRP group (24.7% vs. 9.5%, *p* < 0.001).

**Table 1:** Clinical data grouped by high-sensitivity C-reactive protein.

	All patients	Lower Hs-CRP	Higher Hs-CRP	*p* value
Age, years	64.02 ± 13.20	62.56 ± 12.98	65.25 ± 13.28	0.010
Gender, *n* (%)				0.509
Male	506 (78.60%)	236 (79.70%)	270 (77.60%)	
Female	138 (21.40%)	60 (20.30%)	78 (22.40%)	
Smoking, *n* (%)				0.333
Yes	283 (43.90%)	124 (41.90%)	159 (45.70%)	
No	361 (56.10%)	172 (58.10%)	189 (54.30%)	
Alcohol consumption, *n* (%)				0.364
Yes	408 (63.40%)	182 (61.50%)	226 (64.90%)	
No	236 (36.60%)	114 (38.50%)	122 (35.10%)	
Hypertension, *n* (%)				0.302
Yes	461 (71.60%)	206 (69.60%)	255 (73.30%)	
No	183 (28.40%)	90 (30.40%)	93 (26.70%)	
Diabetes, *n* (%)				0.275
Yes	243 (37.70%)	105 (35.50%)	138 (39.70%)	
No	401 (62.30%)	191 (64.50%)	210 (60.30%)	
Stroke, *n* (%)				0.087
Yes	155 (24.10%)	62 (20.90%)	93 (26.70%)	
No	489 (75.90%)	234 (79.10%)	255 (73.30%)	
Chronic kidney disease, *n* (%)				<0.001
Yes	122 (18.90%)	30 (10.10%)	92 (26.40%)	
No	522 (81.10%)	266 (89.90%)	256 (73.60%)	
Body mass index, kg/m^2^	24.97 ± 3.59	25.04 ± 3.51	24.91 ± 3.66	0.656
Systolic blood pressure, mmHg	130.71 ± 22.58	131.39 ± 21.72	130.13 ± 23.30	0.479
Diastolic blood pressure, mmHg	77.73 ± 13.78	78.58 ± 13.35	77.01 ± 14.11	0.148
Heart rate, bpm	82.42 ± 16.33	79.26 ± 14.25	85.11 ± 17.49	<0.001
White blood cell count, 10^9^/L	10.05 (7.61, 12.67)	9.08 (7.14, 11.83)	10.91 (8.24, 13.65)	<0.001
Hemoglobin, g/L	136.97 ± 22.60	142.18 ± 20.61	132.53 ± 23.28	<0.001
Platelet, ×10^9^/L	220.04 ± 67.60	216.36 ± 59.68	223.17 ± 73.62	0.196
Triglycerides, mmol/L	1.38 (1.02, 1.99)	1.44 (1.02, 2.19)	1.37 (1.02, 1.86)	0.257
Total cholesterol, mmol/L	4.55 ± 1.16	4.61 ± 1.10	4.49 ± 1.21	0.187
LDL-C, mmol/L	2.76 ± 0.87	2.75 ± 0.78	2.77 ± 0.94	0.849
HDL-C, mmol/L	1.14 ± 0.25	1.18 ± 0.26	1.10 ± 0.25	<0.001
Alanine aminotransferase, U/L	33.00 (20.00, 54.00)	31.50 (20.00, 52.00)	34.50 (20.00, 56.25)	0.266
Aspartate aminotransferase, U/L	73.00 (29.00, 176.75)	75.50 (29.00, 188.75)	69.50 (29.00, 161.00)	0.624
Total bilirubin, umol/L	13.50 (9.90, 18.38)	13.80 (10.13, 17.60)	13.10 (9.33, 19.20)	0.504
Albumin, g/L	37.82 ± 4.13	38.93 ± 3.83	36.88 ± 4.15	<0.001
Blood urea nitrogen, mmol/L	5.70 (4.40, 7.50)	5.30 (4.20, 6.68)	6.15 (4.60, 8.60)	<0.001
Creatinine, umol/L	79.00 (66.00, 98.00)	73.50 (62.00, 89.00)	84.00 (69.00, 109.00)	<0.001
eGFR, mL/min/1.73 m^2^	92.73 (68.68, 117.60)	103.67 (80.51, 126.15)	83.86 (58.20, 109.57)	<0.001
Uric acid, umol/L	358.95 ± 115.74	340.60 ± 102.36	374.57 ± 124.03	<0.001
Fasting glucose, mmol/L	6.42 (5.43, 8.31)	6.13 (5.26, 7.90)	6.56 (5.54, 8.65)	0.012
Hemoglobin A1c, %	6.66 ± 1.70	6.46 ± 1.48	6.85 ± 1.86	0.012
Fibrinogen, g/L	3.85 ± 0.99	3.37 ± 0.78	4.27 ± 0.96	<0.001
D-dimer, ug/L	194.50 (89.00, 391.75)	145.00 (69.25, 288.75)	217.50 (118.00, 508.25)	<0.001
Postoperative delirium				<0.001
Yes	114 (17.70%)	28 (9.50%)	86 (24.70%)	
No	530 (82.30%)	268 (90.50%)	262 (75.30%)	

Lower Hs-CRP: Hs-CRP ≤ 4.78 mg/L, Higher Hs-CRP: Hs-CRP > 4.78 mg/L. Hs-CRP, high-sensitivity C-reactive protein; LDL-C, low-density lipoprotein cholesterol; HDL-C, high-density lipoprotein cholesterol; eGFR, estimated glomerular filtration rate.

According to the results in Table 2, there were significant differences in multiple clinical indicators between the POD group and the non-POD group. The average age of patients in the POD group was significantly higher than that in the non-POD group (74.61 years vs. 61.74 years, *p* < 0.001), and the proportion of females was also higher (36.80% vs. 18.10%, *p* < 0.001). Regarding lifestyle factors, the POD group had lower rates of smoking (39.50% vs. 59.60%, *p* < 0.001) and alcohol consumption (21.90% vs. 39.80%, *p* < 0.001). Comorbidity analysis showed that the prevalence of hypertension (86.80% vs. 68.30%, *p* < 0.001), diabetes (49.10% vs. 35.30%, *p* = 0.006), stroke history (40.40% vs. 20.60%, *p* < 0.001), and CKD (50.00% vs. 12.30%, *p* < 0.001) was significantly higher in the POD group. In terms of biomarkers, the POD group showed significantly higher levels of SBP, BUN, creatinine, uric acid, HbA1c, D-dimer, fibrinogen, and Hs-CRP compared to the non-POD group, while DBP, hemoglobin, ALT, AST, total bilirubin, albumin, and eGFR levels were significantly lower in the POD group (*p* < 0.05).

**Table 2:** Clinical data grouped by postoperative delirium.

	Non-POD	POD	*p* value
*N*	530 (82.30%)	114 (17.70%)	
Age, years	61.74 ± 12.78	74.61 ± 9.44	<0.001
Gender, *n* (%)			<0.001
Male	434 (81.90%)	72 (63.20%)	
Female	96 (18.10%)	42 (36.80%)	
Smoking, *n* (%)			<0.001
Yes	316 (59.60%)	45 (39.50%)	
No	214 (40.40%)	69 (60.50%)	
Alcohol consumption, *n* (%)			<0.001
Yes	211 (39.80%)	25 (21.90%)	
No	319 (60.20%)	89 (78.10%)	
Hypertension, *n* (%)			<0.001
Yes	362 (68.30%)	99 (86.80%)	
No	168 (31.70%)	15 (13.20%)	
Diabetes, *n* (%)			0.006
Yes	187 (35.30%)	56 (49.10%)	
No	343 (64.70%)	58 (50.90%)	
Stroke, *n* (%)			<0.001
Yes	109 (20.60%)	46 (40.40%)	
No	421 (79.40%)	68 (59.60%)	
Chronic kidney disease, *n* (%)			<0.001
Yes	65 (12.30%)	57 (50.00%)	
No	465 (87.70%)	57 (50.00%)	
Body mass index, kg/m^2^	25.06 ± 3.52	24.49 ± 3.91	0.146
Systolic blood pressure, mmHg	129.70 ± 22.35	135.40 ± 23.16	0.014
Diastolic blood pressure, mmHg	78.31 ± 13.91	75.06 ± 12.87	0.022
Heart rate, bpm	81.95 ± 16.04	84.61 ± 17.54	0.114
White blood cell count, 10^9^/L	10.19 (7.71, 12.81)	9.84 (7.41, 12.11)	0.414
Hemoglobin, g/L	140.31 ± 21.04	121.45 ± 23.18	<0.001
Platelet, ×10^9^/L	219.19 ± 66.32	223.96 ± 73.46	0.495
Triglycerides, mmol/L	1.40 (1.03, 2.02)	1.31 (0.97, 1.88)	0.165
Total cholesterol, mmol/L	4.53 ± 1.10	4.61 ± 1.40	0.567
LDL-C, mmol/L	2.74 ± 0.83	2.84 ± 1.02	0.316
HDL-C, mmol/L	1.14 ± 0.25	1.15 ± 0.26	0.654
Alanine aminotransferase, U/L	36.00 (22.00, 56.00)	22.00 (15.00, 38.00)	<0.001
Aspartate aminotransferase, U/L	85.00 (31.00, 192.00)	41.00 (22.00, 104.00)	<0.001
Total bilirubin, umol/L	13.80 (10.05, 18.80)	11.80 (8.40, 15.75)	0.001
Albumin, g/L	38.30 ± 3.96	35.59 ± 4.20	<0.001
Blood urea nitrogen, mmol/L	5.40 (4.20, 6.90)	7.70 (5.60, 11.50)	<0.001
Creatinine, umol/L	76.00 (64.00, 92.00)	104.00 (81.00, 148.50)	<0.001
eGFR, mL/min/1.73 m^2^	98.73 (77.00, 123.24)	59.67 (36.42, 84.64)	<0.001
Uric acid, umol/L	349.59 ± 109.93	402.47 ± 131.66	<0.001
Fasting glucose, mmol/L	6.37 (5.42, 8.07)	6.83 (5.40, 9.27)	0.117
Hemoglobin A1c, %	6.52 ± 1.55	7.33 ± 2.17	0.002
Fibrinogen, g/L	3.76 ± 0.96	4.28 ± 0.99	<0.001
D-dimer, ug/L	168.00 (78.50, 333.50)	309.00 (174.50, 694.50)	<0.001
Hs-CRP	4.41 (1.05, 18.82)	15.40 (4.51, 55.55)	<0.001

LDL-C, low-density lipoprotein cholesterol; HDL-C, high-density lipoprotein cholesterol; eGFR, estimated glomerular filtration rate; Hs-CRP, high-sensitivity C-reactive protein.

### 3.2 Logistic regression analysis of high-sensitivity C-reactive protein and postoperative delirium

In the univariate logistic regression analysis shown in Table 3, multiple variables were significantly associated with the occurrence of POD. Specifically, age (OR = 1.101, *p* < 0.001), female gender (OR = 2.637, *p* < 0.001), smoking (OR = 2.264, *p* < 0.001), alcohol consumption (OR = 2.355, *p* < 0.001), hypertension (OR = 3.063, *p* < 0.001), diabetes (OR = 1.771, *p* = 0.006), stroke (OR = 2.613, *p* < 0.001), CKD (OR = 7.154, *p* < 0.001), SBP (OR = 1.011, *p* = 0.015), DBP (OR = 0.982, *p* = 0.023), BUN (OR = 1.117, *p* < 0.001), creatinine (OR = 1.004, *p* < 0.001), uric acid (OR = 1.004, *p* < 0.001), and HbA1c (OR = 1.276, *p* < 0.001) all showed significant effects. Additionally, hemoglobin (OR = 0.964, *p* < 0.001), AST (OR = 0.998, *p* = 0.011), total bilirubin (OR = 0.959, *p* = 0.008), and albumin (OR = 0.851, *p* < 0.001) were negatively associated with POD. Furthermore, Hs-CRP (OR = 1.007, *p* = 0.001), its logarithmic transformation (Log_10_Hs-CRP, OR = 1.972, *p* < 0.001), its standardized value (standardized Hs-CRP, OR = 1.335, *p* = 0.001), and the higher Hs-CRP group (OR = 3.142, *p* < 0.001) were all closely associated with an increased risk of POD.

**Table 3:** The univariate logistic regression analysis of postoperative delirium.

	OR (95% CI)	*p* value
Age	1.101 (1.077, 1.125)	<0.001
Female	2.637 (1.698, 4.095)	<0.001
Smoking	2.264 (1.497, 3.424)	<0.001
Alcohol consumption	2.355 (1.462, 3.792)	<0.001
Hypertension	3.063 (1.727, 5.433)	<0.001
Diabetes	1.771 (1.178, 2.664)	0.006
Stroke	2.613 (1.701, 4.014)	<0.001
Chronic kidney disease	7.154 (4.563, 11.215)	<0.001
Body mass index	0.955 (0.897, 1.016)	0.146
Systolic blood pressure	1.011 (1.002, 1.020)	0.015
Diastolic blood pressure	0.982 (0.967, 0.998)	0.023
Heart rate	1.010 (0.998, 1.022)	0.115
White blood cell count	0.979 (0.930, 1.031)	0.426
Hemoglobin	0.964 (0.955, 0.973)	<0.001
Platelet	1.001 (0.998, 1.004)	0.494
Triglycerides	0.943 (0.780, 1.140)	0.542
Total cholesterol	1.060 (0.893, 1.257)	0.504
Low-density lipoprotein cholesterol	1.141 (0.910, 1.430)	0.253
High-density lipoprotein cholesterol	1.199 (0.543, 2.646)	0.653
Alanine aminotransferase	1.000 (0.998, 1.002)	0.961
Aspartate aminotransferase	0.998 (0.996, 0.999)	0.011
Total bilirubin	0.959 (0.929, 0.989)	0.008
Albumin	0.851 (0.807, 0.897)	<0.001
Blood urea nitrogen	1.117 (1.069, 1.168)	<0.001
Creatinine	1.004 (1.002, 1.006)	<0.001
eGFR	0.973 (0.967, 0.979)	<0.001
Uric acid	1.004 (1.002, 1.005)	<0.001
Fasting glucose	1.039 (0.998, 1.082)	0.065
Hemoglobin A1c	1.276 (1.125, 1.448)	<0.001
Fibrinogen	1.658 (1.352, 2.033)	<0.001
D-dimer	1.000 (1.000, 1.000)	0.352
Hs-CRP	1.007 (1.003, 1.011)	0.001
Log_10_Hs-CRP	1.972 (1.498, 2.595)	<0.001
Standardized Hs-CRP	1.335 (1.124, 1.585)	0.001
Lower Hs-CRP	Ref	
Higher Hs-CRP	3.142 (1.985, 4.973)	<0.001

eGFR, estimated glomerular filtration rate; Hs-CRP, high-sensitivity C-reactive protein; OR, odd ratio; CI, confidence interval.

In the multivariable logistic regression analysis presented in Table 4, Hs-CRP and its various forms were significantly associated with the risk of POD across different adjustment models. Specifically, in Model 1, which adjusts for age and gender, Hs-CRP (OR = 1.008, *p* = 0.001), Log_10_Hs-CRP (OR = 1.920, *p* < 0.001), standardized Hs-CRP (OR = 1.399, *p* = 0.001), and the higher Hs-CRP group (OR = 2.930, *p* < 0.001) were all significantly associated with an increased risk of POD. In Model 2, which further adjusted for smoking, alcohol consumption, hypertension, diabetes, stroke, and CKD, Hs-CRP (OR = 1.006, *p* = 0.012), Log_10_Hs-CRP (OR = 1.684, *p* = 0.001), standardized Hs-CRP (OR = 1.312, *p* = 0.012), and the higher Hs-CRP group (OR = 2.417, *p* = 0.001) maintained statistical significance. In Model 3, which included additional adjustments for SBP, DBP, hemoglobin, AST, total bilirubin, albumin, BUN, creatinine, eGFR, uric acid, HbA1c, and fibrinogen, Hs-CRP (OR = 1.007, *p* = 0.037), Log_10_Hs-CRP (OR = 1.645, *p* = 0.018), standardized Hs-CRP (OR = 1.346, *p* = 0.037), and the higher Hs-CRP group (OR = 2.243, *p* = 0.003) remained significantly associated with POD.

**Table 4:** Multivariable logistic regression analysis of high-sensitivity C-reactive protein and postoperative delirium.

	Model 1		Model 2		Model 3	
	OR (95% CI)	*p* value	OR (95% CI)	*p* value	OR (95% CI)	*p* value
Hs-CRP	1.008 (1.003, 1.012)	0.001	1.006 (1.001, 1.011)	0.012	1.007 (1.000, 1.013)	0.037
Log_10_Hs-CRP	1.920 (1.415, 2.604)	<0.001	1.684 (1.223, 2.317)	0.001	1.645 (1.088, 2.487)	0.018
Standardized Hs-CRP	1.399 (1.145, 1.710)	0.001	1.312 (1.060, 1.623)	0.012	1.346 (1.018, 1.779)	0.037
Lower Hs-CRP	Ref		Ref		Ref	
Higher Hs-CRP	2.930 (1.788, 4.799)	<0.001	2.417 (1.442, 4.050)	0.001	2.243 (1.327, 3.793)	0.003

Model 1 was adjusted for age and gender; Model 2 was adjusted for age, gender, smoking, alcohol consumption, hypertension, diabetes, stroke, and chronic kidney disease; Model 3 was adjusted for all variables in Model 2, as well as systolic blood pressure, diastolic blood pressure, hemoglobin, aspartate aminotransferase, total bilirubin, albumin, blood urea nitrogen, creatinine, estimated glomerular filtration rate, uric acid, hemoglobin A1c, and fibrinogen. Hs-CRP, high-sensitivity C-reactive protein; OR, odd ratio; CI, confidence interval.

### 3.3 Multivariable stratified association between high-sensitivity C-reactive protein and postoperative delirium

In the multivariable stratified analysis shown in Table 5, Hs-CRP was significantly associated with the risk of POD in several stratified variables. Specifically, in patients aged ≥65 years, higher Hs-CRP was significantly associated with POD (OR = 1.914, *p* = 0.024). In the gender stratification, the association between Hs-CRP and POD was significant in female patients (OR = 4.753, *p* = 0.002). Among the hypertension strata, the association was more pronounced in patients without hypertension (OR = 60.862, *p* = 0.001), while it also remained significant in those with hypertension (OR = 1.793, *p* = 0.041). In the diabetes stratification, Hs-CRP was significantly associated with POD in both non-diabetic patients (OR = 1.634, *p* = 0.001) and diabetic patients (OR = 3.349, *p* = 0.004). In the stroke stratification, significant associations were observed in both patients without a history of stroke (OR = 2.053, *p* = 0.033) and those with a history of stroke (OR = 2.655, *p* = 0.024). For the CKD stratification, Hs-CRP was significantly associated with POD in patients without CKD (OR = 1.645, *p* < 0.001).

**Table 5:** Multivariable stratified association between Hs-CRP and postoperative delirium.

	Lower Hs-CRP	Higher Hs-CRP	*p* value
	OR (95% CI)	OR (95% CI)	
Age			
<65 years	Ref	4.262 (0.646, 28.126)	0.132
≥65 years	Ref	1.914 (1.088, 3.367)	0.024
Gender			
Male	Ref	1.268 (0.604, 2.659)	0.530
Female	Ref	4.753 (1.780, 12.691)	0.002
Hypertension			
Yes	Ref	1.793 (1.025, 3.136)	0.041
No	Ref	60.862 (5.750, 644.157)	0.001
Diabetes			
Yes	Ref	3.349 (1.471, 7.625)	0.004
No	Ref	1.634 (1.219, 2.190)	0.001
Stroke			
Yes	Ref	2.655 (1.139, 6.188)	0.024
No	Ref	2.053 (1.058, 3.983)	0.033
Chronic kidney disease			
Yes	Ref	1.255 (0.408, 3.855)	0.692
No	Ref	1.645 (1.277, 2.118)	<0.001

The multivariable stratified association was adjusted for age, gender, smoking, alcohol consumption, hypertension, diabetes, stroke, chronic kidney disease, systolic blood pressure, diastolic blood pressure, hemoglobin, aspartate aminotransferase, total bilirubin, albumin, blood urea nitrogen, creatinine, estimated glomerular filtration rate, uric acid, hemoglobin A1c, and fibrinogen except for the variable that was used for stratification. Hs-CRP, high-sensitivity C-reactive protein; OR, odd ratio; CI, confidence interval.

### 3.4 Predictive value of high-sensitivity C-reactive protein for postoperative delirium across populations

**Figure 1:**
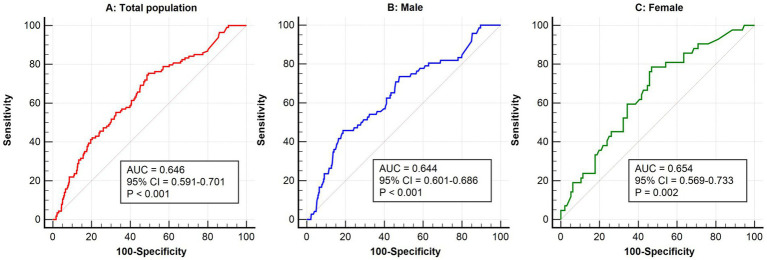
ROC analysis of high-sensitivity C-reactive protein in predicting postoperative delirium in the general population **(A)**, males **(B)**, and females **(C)**. ROC, receiver operator characteristic; AUC, area under the curve; CI, confidence interval.

Figure 1 presented the ROC curve analysis results of Hs-CRP in predicting the risk of POD. In the total population, the AUC for Hs-CRP was 0.646 (95% CI: 0.591–0.701, *p* < 0.001), indicating a significant predictive ability in the overall population. In the gender-stratified analysis, the AUC for male patients was 0.644 (95% CI: 0.601–0.686, *p* < 0.001), and for female patients, it was 0.654 (95% CI: 0.569–0.733, *p* = 0.002), showing that Hs-CRP was significantly associated with the occurrence of POD in both males and females.

### 3.5 Linear association between high-sensitivity C-reactive protein and postoperative delirium risk

**Figure 2:**
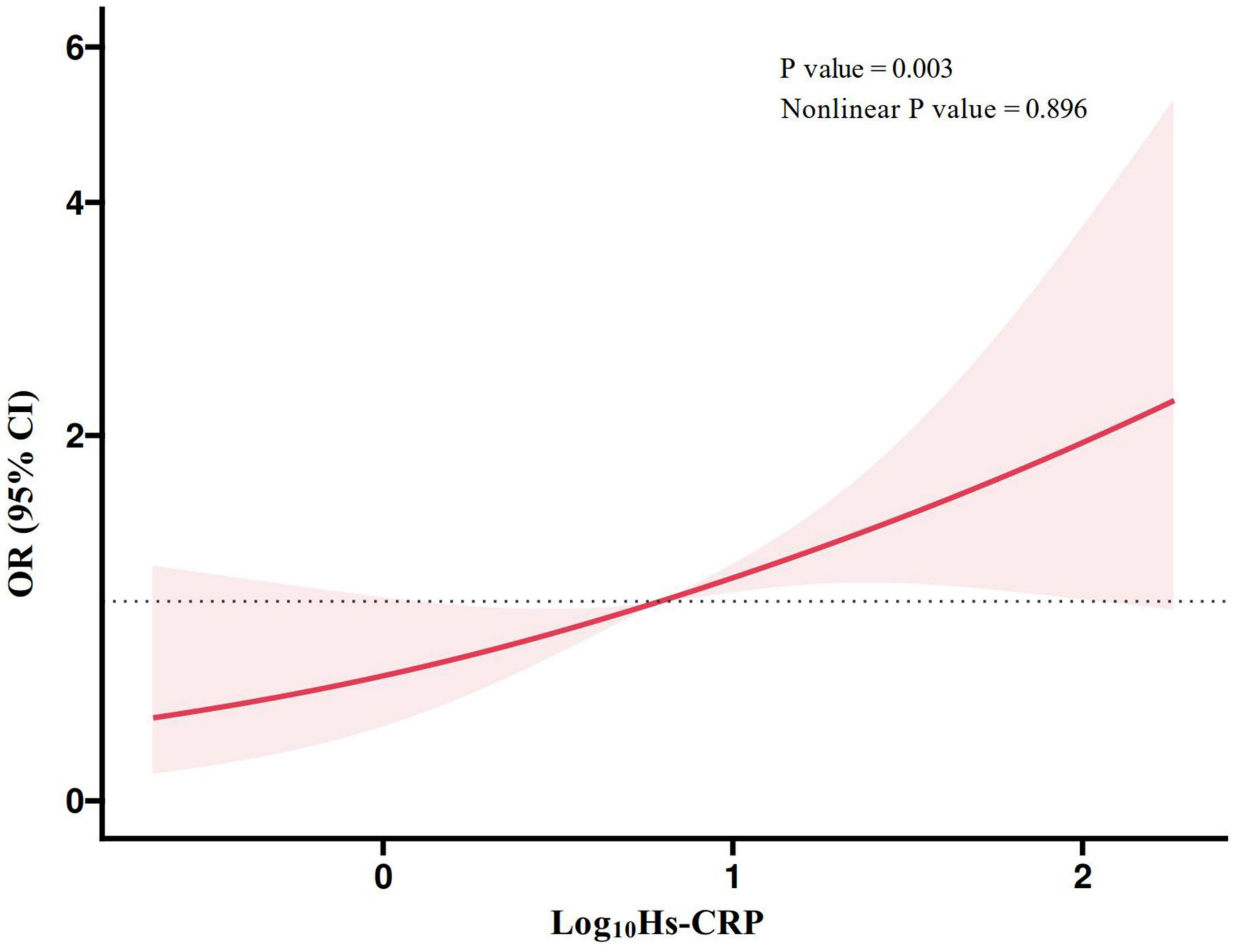
RCS plot of the linear association between high-sensitivity C-reactive protein and postoperative delirium. Hs-CRP, high-sensitivity C-reactive protein; RCS, restricted cubic spline; OR, odd ratio; CI, confidence interval.

Figure 2 presented the RCS plot of the linear association between the Log_10_Hs-CRP and the risk of POD. The results showed a significant positive association between Log_10_Hs-CRP and the risk of POD (*p* = 0.003), and this relationship was linear (nonlinear *p* value = 0.896). This indicated that as Log_10_Hs-CRP increased, the risk of POD gradually rised.

## 4 Discussion

This study investigated the relationship between preoperative Hs-CRP levels and the risk of POD following general anesthesia. The results showed a significant positive association between elevated Hs-CRP levels and the occurrence of POD. Multivariate logistic regression analysis indicated that Hs-CRP, along with its logarithmic and standardized forms, and particularly the higher Hs-CRP group, were significantly associated with increased POD risk. Furthermore, stratified analyses revealed that the association between Hs-CRP and POD risk was more pronounced in certain subgroups, particularly in patients aged ≥65 years, female patients, and those with or without hypertension, diabetes, or stroke history, with an especially strong association observed in patients without CKD. ROC curve analysis indicated that Hs-CRP has moderate predictive value for POD in the overall population, with significant predictive capabilities in both male and female subgroups. RCS analysis further supported a linear positive association between Log_10_Hs-CRP and POD risk, underscoring the importance of Hs-CRP as a potential marker for POD risk assessment.

Our findings are consistent with previous studies that consider CRP as an inflammatory marker associated with neurocognitive impairment, including POD. For example, in a prospective cohort study involving 547 patients aged 70 and older undergoing major non-cardiac surgery, Vasunilashorn et al. found a strong association between CRP and POD, though this association was influenced by genetic factors (11). In addition, in a retrospective study involving 643 colorectal cancer patients, Sun et al. found that a high CRP level on postoperative day 1 was an independent risk factor for POD (20). This suggests that postoperative CRP levels can serve as an independent predictor of POD in colorectal cancer patients, aiding clinicians in the early identification and intervention for high-risk POD patients among elderly colorectal cancer patients. Furthermore, in a nested case–control study involving 566 patients aged 70 and older undergoing major non-cardiac surgery, Dillon et al. confirmed that elevated preoperative and postoperative CRP levels were closely associated with the occurrence of delirium, which suggests that CRP can serve as an early predictive marker for postoperative delirium in elderly surgical patients, aiding in the identification of high-risk individuals and the implementation of preventive measures (13). Moreover, Lozano-Vicario et al. included 60 patients aged 75 and older undergoing hip fracture repair surgery and found that although elevated CRP levels were associated with POD in elderly hip fracture patients, pre-existing cognitive impairment and infections were more significant risk factors (21). Furthermore, an observational study involving 314 patients from the SuDoCo trial found that elevated pre-operative CRP levels were independently associated with POD, but showed no significant association with post-operative neurocognitive disorder, which suggests that pre-operative CRP levels could serve as a predictive marker for POD risk, aiding clinicians in risk stratification among elderly patients and providing a basis for pre-operative interventions to reduce the occurrence of POD (22). In addition, another single-center cross-sectional study also found that higher levels of platelet-to-lymphocyte ratio (PLR), neutrophil-to-lymphocyte ratio (NLR), and systemic inflammation index (SII) were independently associated with an increased risk of POD, suggesting that inflammation may play a crucial role in the development of POD (23). However, these studies primarily focus on traditional CRP, while research on Hs-CRP, a more sensitive measurement indicator, is limited in this field. The advantage of our study lies in the use of Hs-CRP, which enables the detection of low-level inflammation that may lead to POD, particularly in subclinical states. Additionally, the cross-sectional design of our study allowed us to adjust for multiple variables, providing a more comprehensive assessment of the relationship between Hs-CRP and POD. Through stratified analysis, we further revealed that Hs-CRP may serve as a powerful risk indicator for POD within specific subgroups, expanding the current literature on the role of Hs-CRP in POD risk across different patient populations. Furthermore, we not only confirmed the predictive value of Hs-CRP for POD in the overall population and among different gender groups but also established a positive linear correlation between Hs-CRP and POD. This provides new insights and theoretical support for the use of Hs-CRP in early pre-operative screening and management of POD.

However, although growing evidence in recent years indicates a close link between inflammation and POD, the pathophysiological mechanisms underlying this relationship remain incompletely understood (24). The potential mechanism by which elevated Hs-CRP contributes to POD may relate to its role as a marker of systemic inflammation. The inflammation induced by surgery and anesthesia can activate microglia, increase the release of pro-inflammatory cytokines, disrupt the blood–brain barrier, and subsequently trigger neuroinflammation, ultimately affecting cognitive function (25). Hs-CRP may reflect a state that exacerbates these inflammatory pathways. Research has shown that inflammation plays a key role in neurodegenerative diseases and cognitive impairment, supporting the hypothesis that elevated Hs-CRP increases susceptibility to POD through inflammation-driven neurobiological changes (26). Additionally, elevated Hs-CRP may promote POD by inducing oxidative stress. The inflammatory response triggered by surgery and anesthesia can increase the production of reactive oxygen species, heightening oxidative stress levels, which in turn damages neuronal function and further exacerbates neuroinflammatory responses (27, 28). Moreover, elevated Hs-CRP may be associated with the migration of peripheral immune cells into the central nervous system (29). Inflammatory responses may alter blood–brain barrier permeability, making it easier for peripheral immune cells (such as monocytes and neutrophils) to enter brain tissue, and the infiltration of these peripheral immune cells into the central nervous system can worsen the local inflammatory environment, impair brain function, and increase the risk of POD (29–31). However, further research is needed to explore the underlying biological mechanisms of these associations.

Although this study reveals important findings, it has some limitations. Firstly, the average age of patients in the high Hs-CRP group was significantly higher than that in the low Hs-CRP group, and age is a known major risk factor for POD. This difference could act as a confounding factor, interfering with the independent association between Hs-CRP and POD risk. Secondly, the prevalence of CKD was significantly higher in the high Hs-CRP group. CKD can influence Hs-CRP levels through inflammation and metabolic disorders and may independently increase the risk of POD, posing challenges to the independent evaluation of the study results. Additionally, patients in the high Hs-CRP group exhibited significant differences in various physiological and metabolic indicators, including elevated heart rate, white blood cell count, BUN, creatinine, uric acid, fasting glucose, HbA1c, fibrinogen, and D-dimer levels, as well as reduced hemoglobin, HDL-C, albumin, and eGFR levels. These abnormalities may reflect inflammatory states, nutritional status, and systemic metabolic alterations, potentially contributing to the risk of POD and complicating the interpretation of results. Moreover, although the group differences were statistically significant, the clinical relevance of certain indicators may be limited; for example, some biochemical differences may not directly indicate substantial changes in POD risk. Therefore, future studies should further validate these findings and optimize study designs, such as through matching or stratified analyses, to better control potential confounding factors and improve the scientific rigor and clinical applicability of the research. Secondly, the cross-sectional design restricts our ability to determine a causal relationship between elevated Hs-CRP and POD. This limitation underscores the importance of longitudinal studies to clarify the temporal sequence and causative mechanisms underlying this association. Thirdly, while the sample size is relatively large, this is a single-center study, which may limit the generalizability of the results. Expanding this research to multi-center cohorts would strengthen the external validity of the findings. Additionally, relying solely on Hs-CRP as an inflammatory marker may not fully capture the underlying inflammatory processes associated with POD. Incorporating a panel of inflammatory biomarkers could provide a more comprehensive understanding of the inflammatory pathways involved in POD. Future research should consider longitudinal designs, multi-center involvement, and additional biomarkers to further validate and expand upon these findings.

## 5 Conclusion

In conclusion, this study indicates that elevated pre-operative Hs-CRP levels are significantly associated with an increased risk of POD following general anesthesia. These findings suggest that Hs-CRP could serve as a valuable marker for POD risk assessment in clinical settings. Early identification of high-risk patients using Hs-CRP levels could facilitate timely preventive measures and tailored interventions, potentially reducing the incidence of POD. Clinicians may incorporate Hs-CRP testing into pre-operative evaluations, particularly for high-risk patients, to identify and manage those most susceptible to POD. Future research should focus on exploring the mechanisms by which Hs-CRP contributes to POD and on investigating interventions that could mitigate this risk to improve postoperative outcomes in surgical patients. Additionally, further studies should assess the cost-effectiveness and feasibility of incorporating Hs-CRP testing into routine pre-operative workflows.

## Data Availability

The original contributions presented in the study are included in the article/supplementary material, further inquiries can be directed to the corresponding author.
